# Enhanced meningeal lymphatic drainage ameliorates lipopolysaccharide-induced brain injury in aged mice

**DOI:** 10.1186/s12974-024-03028-4

**Published:** 2024-01-30

**Authors:** Hongquan Dong, Xiaonan Dai, Yin Zhou, Chonglong Shi, Piplu Bhuiyan, Zhaochu Sun, Nana Li, Wenjie Jin

**Affiliations:** 1 Department of Anesthesiology, Jiangsu Province Hospital, The First Affiliated Hospital of Nanjing Medical University, Nanjing, 210029 China; 2 Department of Obstetrics, Nanjing Women and Children’s Healthcare Hospital, Women’s Hospital of Nanjing Medical University, Nanjing, 210004 China

**Keywords:** Sepsis-associated encephalopathy, Meningeal lymphatics, Lymphatic endothelial cells, VEGF-C, Neuroinflammation

## Abstract

**Background:**

Sepsis-associated encephalopathy (SAE) is an acute cerebral dysfunction caused by sepsis. Neuroinflammation induced by sepsis is considered a potential mechanism of SAE; however, very little is known about the role of the meningeal lymphatic system in SAE.

**Methods:**

Sepsis was established in male C57BL/6J mice by intraperitoneal injection of 5 mg/kg lipopolysaccharide, and the function of meningeal lymphatic drainage was assessed. Adeno-associated virus 1-vascular endothelial growth factor C (AAV1-VEGF-C) was injected into the cisterna magna to induce meningeal lymphangiogenesis. Ligation of deep cervical lymph nodes (dCLNs) was performed to induce pre-existing meningeal lymphatic dysfunction. Cognitive function was evaluated by a fear conditioning test, and inflammatory factors were detected by enzyme-linked immunosorbent assay.

**Results:**

The aged mice with SAE showed a significant decrease in the drainage of OVA-647 into the dCLNs and the coverage of the Lyve-1 in the meningeal lymphatic, indicating that sepsis impaired meningeal lymphatic drainage and morphology. The meningeal lymphatic function of aged mice was more vulnerable to sepsis in comparison to young mice. Sepsis also decreased the protein levels of caspase-3 and PSD95, which was accompanied by reductions in the activity of hippocampal neurons. Microglia were significantly activated in the hippocampus of SAE mice, which was accompanied by an increase in neuroinflammation, as indicated by increases in interleukin-1 beta, interleukin-6 and Iba1 expression. Cognitive function was impaired in aged mice with SAE. However, the injection of AAV1-VEGF-C significantly increased coverage in the lymphatic system and tracer dye uptake in dCLNs, suggesting that AAV1-VEGF-C promotes meningeal lymphangiogenesis and drainage. Furthermore, AAV1-VEGF-C reduced microglial activation and neuroinflammation and improved cognitive dysfunction. Improvement of meningeal lymphatics also reduced sepsis-induced expression of disease-associated genes in aged mice. Pre-existing lymphatic dysfunction by ligating bilateral dCLNs aggravated sepsis-induced neuroinflammation and cognitive impairment.

**Conclusion:**

The meningeal lymphatic drainage is damaged in sepsis, and pre-existing defects in this drainage system exacerbate SAE-induced neuroinflammation and cognitive dysfunction. Promoting meningeal lymphatic drainage improves SAE. Manipulation of meningeal lymphangiogenesis could be a new strategy for the treatment of SAE.

**Supplementary Information:**

The online version contains supplementary material available at 10.1186/s12974-024-03028-4.

## Background

Sepsis is a potentially fatal organ impairment caused by immunological dysfunction in response to infection [1]. Although sepsis can occur in patients of any age, age is a powerful risk factor because the incidence of sepsis in the elderly is ~ 10 times higher than that in younger adults [2]. Thus, the majority of sepsis survivors are > 65 years of age [3]. Sepsis-associated encephalopathy (SAE) is an acute cerebral dysfunction caused by sepsis without direct brain infection. While recovering patients frequently have cognitive impairment, SAE is frequently linked to an increase in healthcare needs and costs [4]. Indeed, ~ 50% of sepsis survivors have long-term cognitive deficits, the incidence of which is higher in older patients [5].

Sepsis-induced neuroinflammation is a plausible explanation of cognitive impairment [6]. The brain’s resident immune cells, known as microglia, are essential for maintaining the homeostatic state of the central nervous system (CNS). The environment of sepsis facilitates the expression of pathogen-activated molecular patterns (PAMPs) and damage-associated molecular patterns (DAMPs), all of which activate microglia to produce cytotoxic factors such as tumor necrosis factor (TNF), interleukin (IL)-6, nitric oxide (NO), reactive oxygen species (ROS), and others [7]. Accumulation of these pro-inflammatory and cytotoxic factors is directly detrimental to neurons. In the event of CNS damage, ineffective clearance of cytotoxic factors has been postulated to prolong neuroinflammation and encourage the development of CNS disease [8].

Recently, lymphatic vessels in the meninges surrounding the CNS have been rediscovered [9]. The drainage of macromolecules, cellular waste, toxic substances, and immune cells from the brain to the deep cervical lymph nodes (dCLNs) is conducted through meningeal lymphatic vessels [10]. Further research has revealed that amyloid-β, extracellular tau, and α-syn are removed from the brain by the meningeal lymphatic system and that its disturbance might encourage the buildup of these neurotoxic substances in the brain and accelerate the progression of neurodegenerative diseases [11–13]. Meningeal lymphatic function is also significantly altered in aged mice and humans [14, 15]. However, the role of the meningeal lymphatic system in SAE in older patients remains ambiguous.

In the present study, we investigated the effects of sepsis on the meningeal lymphatic system and examined whether overexpression of lymphangiogenic vascular endothelial growth factor c (VEGF-C) improves sepsis-induced neuroinflammation and cognitive impairment. In mice with sepsis, we showed that adeno-associated virus (AAV)-VEGF-C administration through the cisterna magna boosted drainage to dCLNs and enhanced meningeal lymphatic vessel function. Furthermore, AAV-VEGF-C reduced microglial activation and neuroinflammation and alleviated sepsis-related cognitive impairment. We also observed that neuroinflammation and cognitive decline after sepsis were worsened by a pre-existing impairment in meningeal lymphatic function. Our findings suggest that the meningeal lymphatics represent a potential target for treating SAE in elderly patients.

## Methods

### Animals

The Institutional Animal Care and Use Committee of Nanjing Medical University approved the study’s animal experimentation protocols (No. 2008037). The National Institutes of Health of the United States Guide for the Care and Use of Laboratory Animals was followed for all animal experiments. We purchased male C57BL/6J mice from Beijing Vital River Laboratory Animal Technology (Beijing, China), including aged (16–18 months) and young (8 weeks) mice. A total of 145 aged mice and 5 young mice were used in our study. The mice were housed under a standard laboratory environment with free access to food and water, a 12-h/12-h day/night cycle, and a constant room temperature of 22 ± 1 °C.

### Lipopolysaccharide (LPS) treatment

Mice were randomly assigned to experimental groups and received a single intraperitoneal injection of either endotoxin-free phosphate buffered saline (PBS) or LPS (5 mg/kg) derived from *Escherichia coli* O55:B5 (Sigma-Aldrich L2880). The LPS was dissolved and diluted in endotoxin-free PBS. The mice were euthanized at specific time points post-injection. Nine aged mice died after LPS injection.

### Intracisternal magna injections

Mice were placed in a stereotactic frame (RWD) following the administration of isoflurane anesthesia. Ophthalmic solution was applied to the eyes to avoid drying after shaving the neck and cleaning it with 70% ethanol. The muscle layers were pulled back, and the atlanto-occipital membrane of the magna cisterna was revealed after making a small (5 mm) skin incision. The volumes of the necessary solutions were injected into the cerebrospinal fluid-filled cisterna magna compartment at a rate of 2 μl/min using a 33G Hamilton syringe. To avoid CSF leakage, the syringe was left in place for 5 min after the injection. After closing the incision, the mice were subcutaneously injected with buprenorphine (0.5 mg/kg) and placed on a heating pad until full recovery. Subsequently, 3 μl of ovalbumin conjugated with Alexa Fluor 647 (OVA-647, 2 mg/ml, Thermo Fisher Scientific) or viral vector (AAV1-CMV-mVEGF-C or AAV1-CMV-eGFP, at 5.00E + 12 v.g./ml, purchased from Shanghai Genechem Co., Ltd.) was administered via this intra-cisternal magna (i.c.m.) injection (Additional file 1).

### Ligation of deep cervical lymph nodes

Mice were anesthetized with isoflurane, and the rostral neck was shaved and disinfected. Subsequently, a 1.5-cm incision was made 5 mm above the clavicle, in the middle. A deep cervical lymph node was observed when the sternocleidomastoid muscle (SCM) was retracted. A 4-0 nylon suture was used to ligate the afferent lymph vessels on each side. Sham-operated animals underwent incision and SCM retracement but were not subjected to ligation. The incision was closed, after which the mice were subcutaneously injected with buprenorphine (0.5 mg/kg) and placed on a heating pad until full recovery.

### Immunofluorescence staining

After anesthesia with 1% sodium pentobarbital (10 μl/g), the mice were transcardially perfused with phosphate buffered saline (PBS) for 2 min and 4% paraformaldehyde (PFA) for 5 min. Meninges were prepared following a protocol previously described, with slight modifications [16]. The skullcap was harvested and fixed in 4% PFA at 4 °C overnight. Place the skullcap, along with PBS, in a 10-cm petri dish positioned under a dissecting microscope. With fine forceps, secure the skullcap at the section covering the olfactory bulbs. Grab the thick portion of the meninges and gently pull it towards the superior sagittal sinus. Along the interior edge of the skullcap, delicately detach the meninges from the skull using fine forceps, completing a full 360º rotation. Grab the meninges at the tip of the transverse sinuses and gently pull them towards the pineal gland to detach them from the bone junction. Seize the meninges from the pineal gland region and gently pull them out of the skullcap. Subsequently, the meninges were placed in a 24-well plate with 500 μl of PBS. The brain and dCLNs were harvested and postfixed in 4% PFA at 4 °C overnight, followed by cryoprotection in 30% sucrose for 3 days at 4 °C, before embedding in OCT. For immunofluorescence staining, dCLNs were sliced into 10-μm-thick sections, and the brains were sliced into 30-μm-thick sections. The brain slices and meninges in 24-well plate were incubated in 300 μl of PBS with 0.5% Triton-X-100 and 5% bovine serum albumin (BSA) for 1 h at room temperature. This blocking step was followed by incubation with appropriate dilutions of primary antibodies in PBS with 0.5% Triton-X-100 and 1% BSA overnight at 4 °C. Following incubation, the tissues were incubated for 2 h at room temperature in 300 μl of secondary antibodies diluted in PBS with 0.5% Triton-X-100 and 1% BSA after being washed with PBS three times for 15 min each. After washed with PBS, the brain slices and meninges were flattened on a glass slide and kept in the dark for 10 min to let them dry. Finally, whole mounts and sections were mounted using mounting media containing 4,6-diamidino-2-phenylindole (Abcam, ab104139). The following primary antibodies were used for immunofluorescence: rabbit anti-Lyve-1 (Abcam, ab14917, 1:500), mouse anti-Lyve-1-eFluor 570 (eBioscience, 41-0443-82, 1:500), rabbit anti-Iba1 (Wako, 019-19741, 1:500), and rabbit anti-NeuN (Abcam, ab177487, 1:300). Goat anti-rabbit Alexa Fluor 594 and goat anti-rabbit Alexa Fluor 488 were used as secondary antibodies (both purchased from Invitrogen).

### Image analysis

Stained sections were obtained by Thunder Imager DMi8 (Leica Microsystem Wetzlar, Germany), and images were captured with constant exposure time, offset, and gain for each staining marker. The area of positive signal was measured using ImageJ (NIH) with grayscale threshold analysis. The minimum and maximum intensity settings for each staining marker remained constant. For the brain sections and meninges, the percentage area of positive signal for OVA-647, Lyve-1 and Iba-1 was calculated by dividing the positive signal area by the total area of the image. Five sections of each brain were quantified to acquire the mean value. The percent area coverage of Lyve-1 was used to determine the coverage of the lymphatic vessels. For the dCLN sections, the dCLN areas in each section were manually delineated and the percentage area of positive signal for OVA-647 was quantified by dividing the positive signal area by the area of the dCLN section. Five sections of each dCLN were quantified to acquire the mean value. For quantification of NeuN, images were imported into ImageJ to calculate the integrated optical density value of the area occupied by NeuN staining. Five sections per mouse were averaged to provide a mean value for each mouse. Each value was expressed relative to the mean value of control group, which was set to 1. All quantitative analyses were performed in a blinded fashion by an experimenter that was unaware of the sample identity.

### Behavioral testing

The mice underwent a fear conditioning test (FCT) as previously described. The clear acrylic FCT chamber, 32 cm long, 25 cm wide, and 25 cm high, included a floor constructed of stainless-steel bars, which was connected to a shock delivery system. The FCT was conducted over 2 days. On day 1, three tone–foot shock pairings (tone: 2000 Hz, 80 dB, 30 s; foot shock: 0.7 mA, 2 s) with an interval of 1 min were applied to each mouse in a test chamber that had been cleaned with 70% alcohol. The mouse was moved out from the test chamber 30 s after administration of the conditioning stimuli. On day 2, the mice were returned to the same chamber for 5 min without the tone or shock. The animals were then euthanized after testing. Software (Xeye Fcs, Beijing Macro Ambition S&T Development Co., Ltd., Beijing, China) was used to record a video and evaluate the freezing behavior, which was identified as a lack of movement. The experiments were performed in a blinded manner with respect to animal genotype and surgical procedure.

### Western blot

RIPA lysis buffer, containing 50 mM Tris, 150 mM NaCl, 1% Triton X-100, 2 mM EDTA, 1.5 μg/ml leupeptin, and 1 mM phenylmethylsulfonyl fluoride, was used to homogenize hippocampal tissues. The lysate was centrifuged for 20 min at 12,000×*g* and 4 °C. The protein concentration was determined using a BCA assay (Thermo Scientific), and 30 μg of protein was loaded into each lane of a modified gel for analysis by sodium dodecyl sulfate–polyacrylamide gel electrophoresis. The proteins were transferred onto polyvinylidene difluoride membranes (Millipore, Bedford, MA, USA), which were then blocked for 1 h with 5% nonfat milk in Tris-buffered saline Tween 20. The blocked membranes were probed overnight with specific primary antibodies diluted in 5% nonfat milk, as instructed by the manufacturer. Rabbit anti-APP (Abcam, ab15372, 1: 500), rabbit anti-occludin (Abcam, ab167161, 1:500), and mouse anti-GAPDH (Proteintech, 1:1,000) were the primary antibodies used. Before exposure to film, the membranes were washed, incubated with secondary antibodies (1:1,000), and then washed and treated with ECL reagent. Image Lab software (Bio-Rad, Richmond, CA, USA) was used to perform the densitometry analysis, and ImageJ was employed to calculate the results.

### Sorting of meningeal LECs

Mice were anesthetized with sodium pentobarbital by intraperitoneal injection and transcradially perfused with ice-cold PBS. Meninges were quickly removed and digested for 12 min at 37 °C in preheated DMEM (Gibco) with 2% FBS (Gibco), 1 mg/ml collagenase VIII, and 0.5 mg/ml DNase I (Sigma-Aldrich). At the end of digestion, 1 ml DMEM with 10% FBS was added to the solution to terminate digestion. The digested meninges were filtered through a 70-μm nylon-mesh filter. Individual samples consisted of cell suspensions pooled from three meninges and washed with ice-cold fluorescence-activated cell sorting (FACS) buffer (PBS, 1 mM EDTA, 1% BSA, pH 7.2). Cells were then centrifuged at 400*g*, and 4 °C for 5 min, resuspended in 400 μl ice-cold FACS buffer with anti-CD45-BB515 (BD Bioscience, 564590, 1:200), anti-CD31-Alexa Fluor 647 (BD Bioscience, 563608, 1:200), and anti-podoplanin-PE (eBioscience, 12-5381-82, 1:200), and incubated for 30 min at 4 °C. Cells were washed in ice-cold FACS and sorted by MoFlo Astrios EQ (Beckman Coulter, Indianapolis, IN, USA) and analyzed by the FlowJo software (version 10, Tree Star, Ashland, OR, USA). The LECs were gated as CD45^−^CD31^+^podoplanin^+^ cells.

### Quantitative real-time PCR (qRT-PCR)

Total RNA was extracted from LECs with Trizol reagent (Invitrogen, USA), and cDNA was generated using a PrimeScript RT Master kit (Tarara, Japan) according to the manufacturer’s instructions. Relative qRT-PCR was performed on a StepOne Real-time PCR Detection System (Foster City, CA, USA) with SYBR Green master mix (Takara, Japan). The 2^−ΔΔCt^ method was used to calculate the relative gene expression. The sequences of all primers were as follows: *Prox1* forward, AGAAGGGTTGACATTGGAGTGA; *Prox1* reverse, TGCGTGTTGCACCACAGAATA; *Focx2* forward, AACCCAACAGCAAACTTTCCC; *Focx2* reverse, GCGTAGCTCGATAGGGCAG; *GAPDH* forward, AGGTCGGTGTGAACGGATTTG; *GAPDH* reverse, TGTAGACCATGTAGTTGAGGTCA.

### Transcriptome analysis

Samples were obtained from two groups: the eGFP + LPS group and the VEGF-C + LPS group. Sample processing and detection were performed by GeneChem (Shanghai, China). The differentially expressed genes in the hippocampal samples from mice in the eGFP + LPS and VEGF-C + LPS groups were further analyzed. KEGG enrichment analyses were performed for the differentially expressed genes.

### ELISA

Each mouse hippocampus was completely pulverized with a tissue grinder while immersed in lysis buffer. After centrifuging the lysate, the supernatant was collected for ELISA. ELISA kits from R&D Systems (Minneapolis, MN, USA) were used to measure the levels of IL-1β and IL-6 in accordance with the instructions provided. The samples were evaluated in duplicate by ELISA.

### Statistical analysis

All data are presented as the mean ± SD, and differences between mean values were assessed by one-way analysis of variance (ANOVA) and Tukey’s multiple comparison test, as well as Student’s *t* test with unpaired two-tailed tests using GraphPad Prism 5. P values < 0.05 were considered significant.

## Results

### Sepsis impairs meningeal lymphatic drainage function in aged mice

**Fig. 1:**
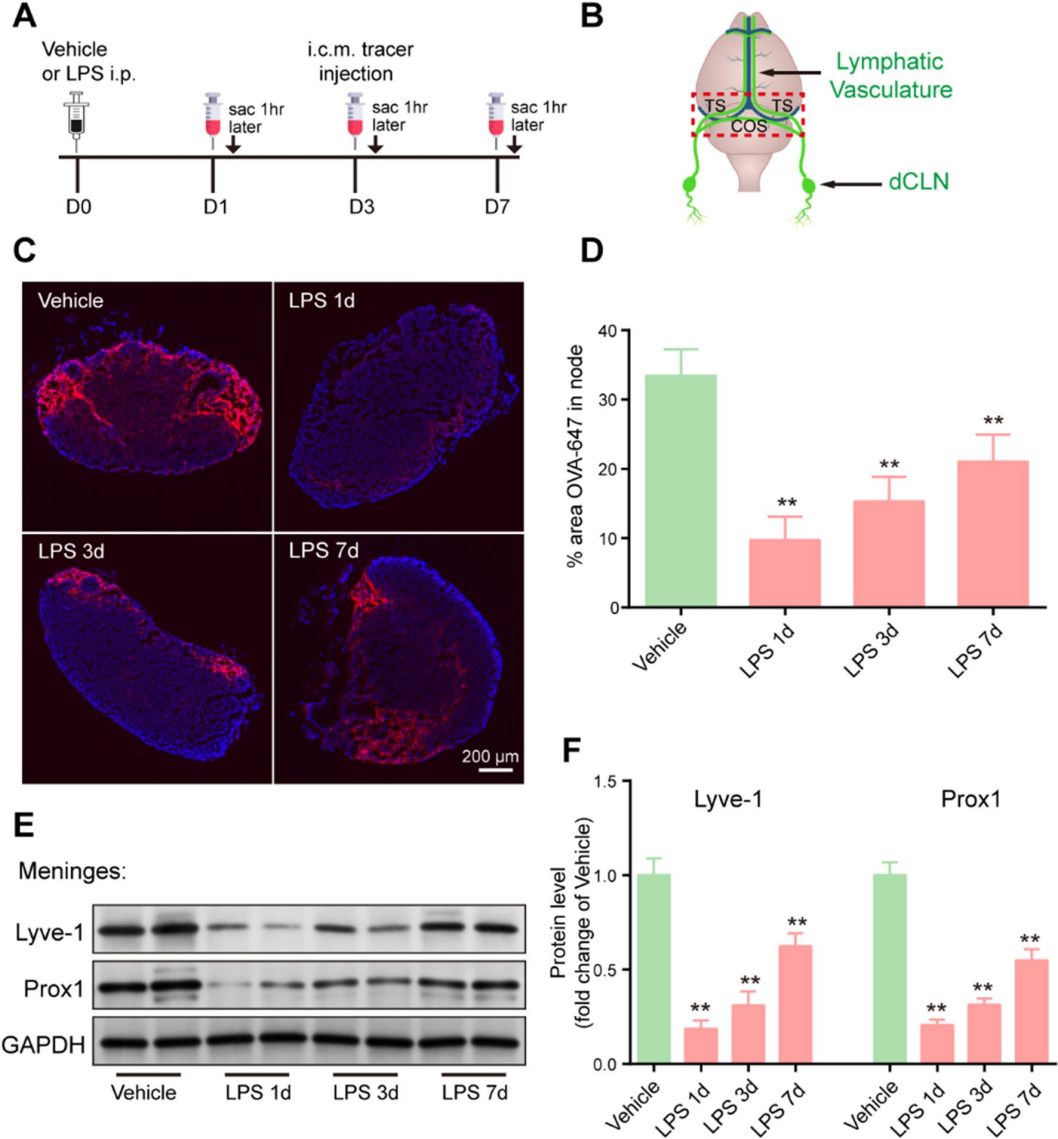
Sepsis impairs meningeal lymphatic drainage function in aged mice.** A** The experimental timeline of LPS treatment, tracer injection into the cisterna magna, and tissue collection (sac). **B** Schematic diagram of the dura mater. transverse sinuses (TS), confluence of sinuses (COS). **C** Representative immunofluorescence images of OVA-647 accumulation in dCLNs. **D** Quantification of the fluorescence intensity of OVA-647 in the dCLNs, n = 6. **E** Representative immunoblot of Lyve-1 and Prox1 proteins in the meninges. **F** Quantification of Lyve-1 and Prox1 proteins in the meninges, n = 4. *P < 0.05, **P < 0.01. All data are expressed as the mean ± SD

To determine whether the function of meningeal lymphatic drainage is affected by sepsis, 5 mg/kg LPS was intraperitoneally injected to establish a model of sepsis, before injecting OVA-647 tracer into the cisterna magna (i.c.m.) at different time points to examine meningeal lymphatic drainage. The mice were killed 1 h after OVA-647 injection, and the dCLNs and meningeal tissues were harvested (Fig. 1A, B). Interestingly, we observed a substantial decrease in the drainage of OVA-647 into the dCLNs in sepsis mice at day 1 (Fig. 1C, D). Additionally, 3 days after LPS injection, the drainage of OVA-647 was still reduced compared to that in the vehicle group (Fig. 1C, D), and meningeal lymphatic drainage function remained significantly damaged until 7 days post-injection (Fig. 1C, D). To investigate whether meningeal lymphatic cells are also damaged by sepsis, we examined the protein levels of Lyve-1, a marker of lymphatic endothelial cells (LECs), and Prox1, a transcription factor that induces lymph angiogenesis, in the meninges. Western blot analysis revealed that sepsis significantly decreased Lyve-1 and Prox1 levels in the meninges compared to those in the vehicle group, especially at 1 day after LPS injection (Fig. 1E, F). Overall, these data indicate that LPS-induced sepsis can result in meningeal lymphatic dysfunction.

### Sepsis damages meningeal lymphatics in aged mice

**Fig. 2:**
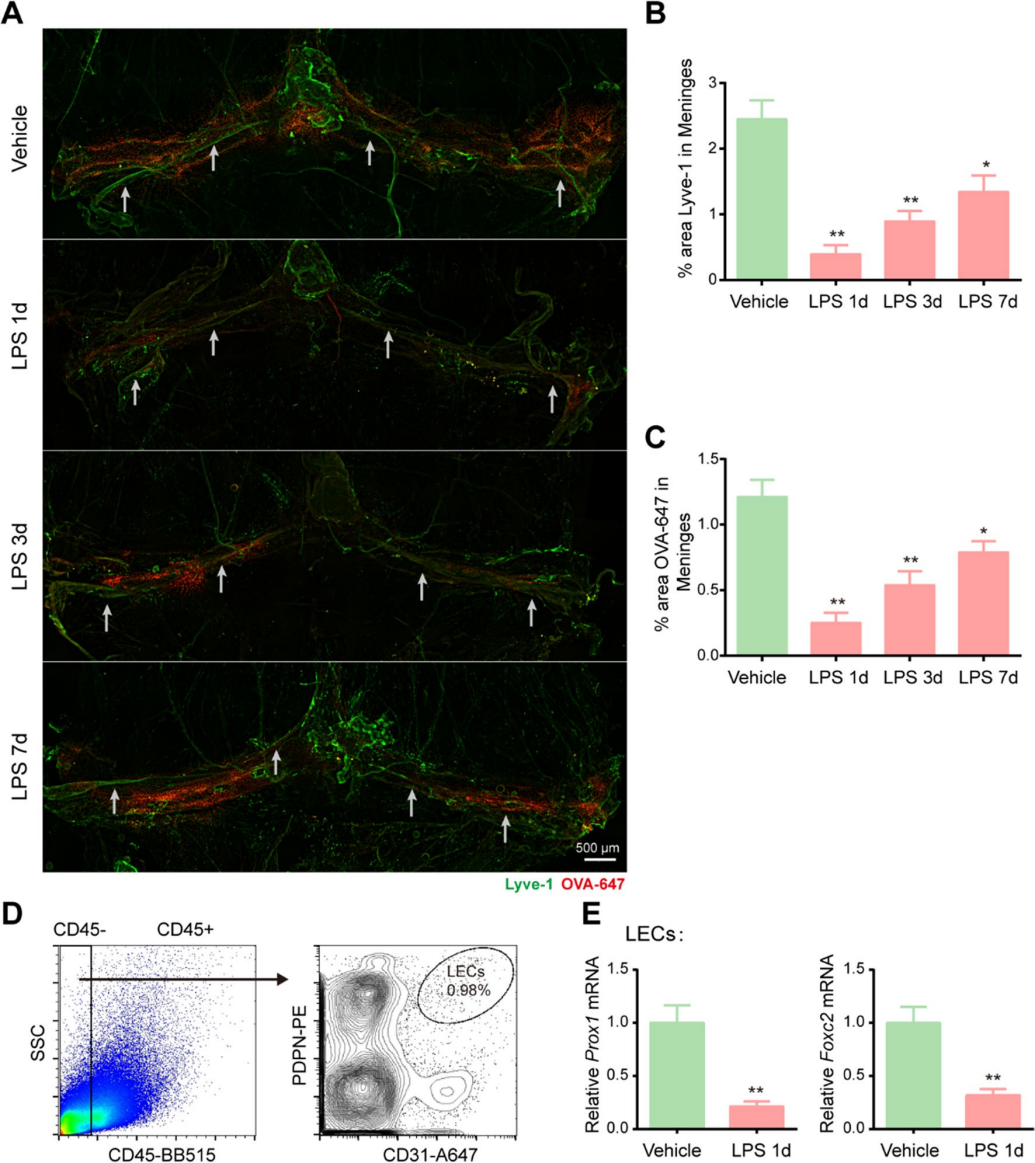
Sepsis damages meningeal lymphatic vasculature morphology in aged mice.** A** Representative immunofluorescence images of meningeal whole-mounts with OVA-647 stained with Lyve-1. **B** Graph depicting the percentage area of Lyve-1 coverage in the meningeal lymphatic vasculature, n = 6. **C** OVA-647 coverage in the meningeal lymphatic vasculature, n = 6. **D** Representative dot and contour plots showing the gating strategy used to isolate meningeal LECs (CD45^−^CD31^+^ podoplanin^+^). **E** Relative mRNA levels of *Prox1* and *Foxc2* in meningeal LECs sorted from mice treated with Vehicle or LPS, n = 4. *P < 0.05, **P < 0.01. All data are expressed as the mean ± SD

In an effort to ascertain whether the functional decline in meningeal lymphatic drainage was also accompanied by changes in meningeal lymphatics, we evaluated the meningeal lymphatic vasculature at diverse time points after LPS injection. We stained the whole meninges with Lyve-1 (green), and injected fluorescent OVA-647 (red) into the cisterna magna at various time points. Analysis of the whole meninges revealed that the tracer was taken up along the transverse sinuses (TS) and confluence of sinuses (COS) (Fig. 2A). We found that the area of Lyve-1 immunofluorescence staining in the meningeal lymphatics were significantly decreased at 1 day post LPS injection (Fig. 2A, B). Additionally, there was an overall decrease in the Lyve-1 percentage area coverage in the meningeal lymphatics. LPS injection also appeared to reduce the diameters of LYVE1-labeled meningeal lymphatic vessels in the TS and COS of the dura mater; white arrows were employed to indicate the position. (Fig. 2A). Consistent with the dysfunction of the meningeal lymphatics, significantly less intracisternally injected tracer was detected in the meningeal lymphatics (Fig. 2A, C). As time progressed, the Lyve-1 and OVA-647 percentage area coverage in the meninges appeared to increase compared to that on the first day post-injection, but it remained lower than that of the vehicle group. Lymphatic valves and smooth muscle cells in lymphatic vessels play an important role in lymphatic fluid transport. Transcription factors, such as Prox1 and Foxc2, are essential to lymphatic valve development and recruitment of smooth muscle cells [17, 18]. To understand the mechanism of meningeal lymphatic dysfunction in SAE, we detected the expression of these genes one day after LPS injection. We found that the mRNA levels of *Prox1* and *Foxc2* in CD31^+^ podoplanin (PDPN)^+^ LECs sorted by FACS were significantly reduced in LPS-injected mice (Fig. 2D, E). Taken together, these findings demonstrate that LPS-induced sepsis causes damage to the drainage of meningeal lymphatics, accompanied by changes in meningeal lymphatic vasculature morphology.

### Meningeal lymphatic function of aged mice is more vulnerable to sepsis

**Fig. 3:**
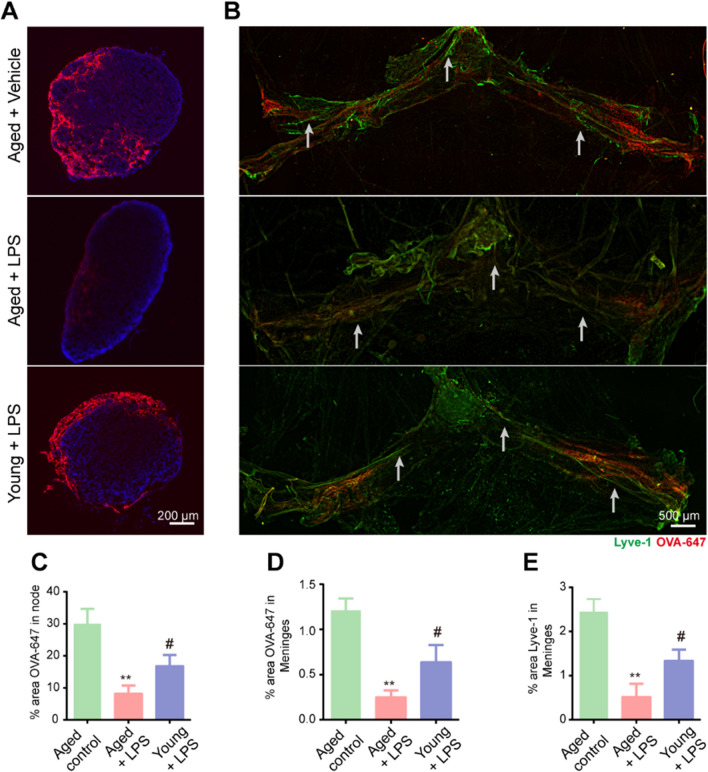
Meningeal lymphatic function in aged mice is more vulnerable to sepsis. **A** Representative immunofluorescence images of OVA-647 accumulation in dCLNs. **B** Representative immunofluorescence images of meningeal whole-mounts of OVA-647 stained with DAPI and Lyve-1. **C** Graph depicting the percentage area of OVA-647 coverage in the dCLNs. **D** Graph depicting the percentage area of OVA-647 coverage in the meningeal lymphatic vasculature. **E** Graph depicting the percentage area of Lyve-1 coverage in the meningeal lymphatic vasculature. n = 5. **P < 0.01, aged control group vs. aged + LPS group, ^#^ P < 0.05, young + LPS group vs. aged + LPS group. All data are expressed as the mean ± SD

Sepsis is an especially critical condition in old age, and the effects of SAE have more devastating consequences among the elderly [19]. However, why seniors are more vulnerable to sepsis and serious complications remains poorly understood. Of note, it has recently been reported that meningeal lymphatic drainage function markedly declines during aging [11], which led us to explore whether the meningeal lymphatic function of aged people is more susceptible to sepsis. Therefore, we divided the mice into three groups: aged + vehicle, aged + LPS, and young + LPS. We injected OVA-647 tracer into the CSF and assessed drainage to the dCLNs at one day after LPS intraperitoneal injection in each group. We observed a significant decrease in the drainage of OVA-647 into the dCLNs (Fig. 3A, C) and less intracisternally injected OVA-647 tracer in the meningeal TS region (Fig. 3B, D) in the aged + LPS group than in the young + LPS group. We also stained the whole meninges with Lyve-1 to evaluate the function of the meningeal lymphatics. The aged + LPS group showed a dramatic decline in the area of the meninges covered by Lyve-1-expressing lymphatic vessels compared to the young + LPS group (Fig. 3B, E). The aged + LPS group also exhibited a reduction in the diameters of LYVE1-labeled meningeal lymphatic vessels in the TS and COS of the dura mater; white arrows were utilized to indicate the position. In summary, these results indicate that the meningeal lymphatic function of aged mice is more likely to be damaged by sepsis than that of young mice.

### Improvement of meningeal lymphatics alleviates sepsis-induced cognitive dysfunction in aged mice

**Fig. 4:**
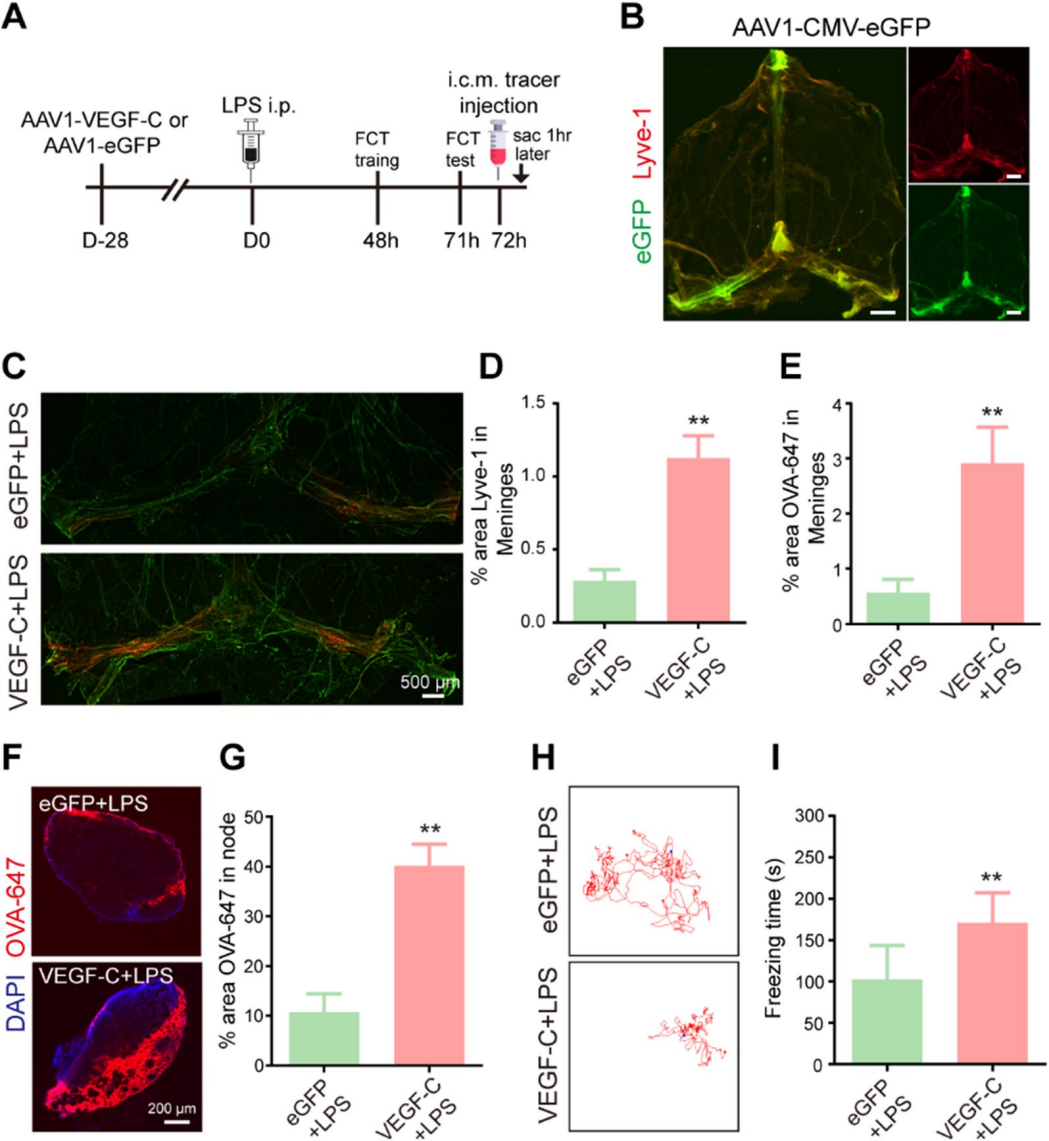
Improvement of meningeal lymphatics alleviates sepsis-induced cognitive dysfunction in aged mice. **A** The experimental timeline of the intracisternal AAV infusion, LPS treatment, behavioral test, tracer injection into the cisterna magna, and tissue collection (sac). **B** Representative images depicting eGFP (green)-labeled AAV1-infected cells surrounding Lyve1 (red)^+^ meningeal lymphatics, scale bars: 1 mm. **C** Representative immunofluorescence images of meningeal whole-mounts of OVA-647 stained with Lyve-1. **D** Graph depicting the percentage area of Lyve-1 coverage in the meningeal lymphatic vasculature, n = 6. **E** Graph depicting the percentage area of OVA-647 coverage in the meningeal lymphatic vasculature, n = 6. **F** Representative immunofluorescent images of OVA-647 accumulation in dCLNs. **G** Graph depicting the percentage area of OVA-647 coverage in the dCLNs, n = 6. **H** Representative trajectory of each group in the FCT. **I** Quantitative of freezing time in the FCT, n = 8. **P < 0.01. All data are expressed as the mean ± SD

To better understand the functional effects of damaged meningeal lymphatic function in sepsis, we next investigated whether improvement of meningeal lymphatics is effective in alleviating sepsis-induced cognitive dysfunction. Importantly, it has been reported that delivery of vascular endothelial growth factor-C (VEGF-C), which binds to vascular endothelial growth factor receptor 3 (VEGFR3), could promote the growth of lymphatic vessels and rejuvenate meningeal lymphatic drainage function [20, 21]. Thus, we employed intracisternal injection of adeno-associated virus (AAV) overexpressing VEGF-C (Fig. 4A), which has previously been confirmed to successfully increase the diameter of the meningeal lymphatic vessels without affecting meningeal blood vessels [22]. Accordingly, we injected enhanced green fluorescent protein (AAV1-CMV-eGFP) as a control into the cisterna magna (Fig. 4A). We observed that AAV-infected cells stably labeled by eGFP were mainly distributed in the TS and COS, which surround the meningeal lymphatics labeled by Lyve-1 (Fig. 4B), in the aged meninges at 28 days after injection. One month later, both groups were subjected to intraperitoneal injection of LPS (Fig. 4A). Unsurprisingly, the aged mice that overexpressed VEGF-C showed a significantly increased area of Lyve-1 immunofluorescence staining in the COS and TS of the dura mater, as well as the coverage area of the meningeal lymphatics (Fig. 4C, D). Furthermore, the mice injected with VEGF-C showed increased drainage of OVA-647 tracer into dCLNs and enhanced meningeal tracer uptake (Fig. 4E–G).

It is universally acknowledged that SAE induces a significant decline in cognitive function. Notably, we also observed that the freezing time of the VEGF-C-treated aged mice was significantly increased compared to that of the eGFP-treated aged mice (Fig. 4H, I), suggesting that VEGF-C can significantly improve cognitive performance. Taken together, these results indicate that improvement of meningeal lymphatics by intracisternal injection of AAV1-VEGF-C alleviates cognitive dysfunction in mice with sepsis.

### Improvement of meningeal lymphatics attenuates sepsis-induced neuronal damage and neuroinflammation in aged mice

Sepsis, which is closely associated with inflammation, can trigger vascular endothelial damage, whereby the blood–brain barrier breaks down and facilitates the entry of peripheral immune cells into the brain, which exacerbates glial cell activation and neuroinflammation [23]. Thus, we next explored whether enhancement of meningeal lymphatics could effectively reduce inflammation and protect hippocampal neurons. To this end, 1 month after viral vector delivery and 3 days after LPS injection, we measured hippocampal neurons and Iba1 immunoreactivity in the hippocampus to assess neuronal damage and establish whether treatment with VEGF-C could improve neuroinflammation and sepsis.

**Fig. 5:**
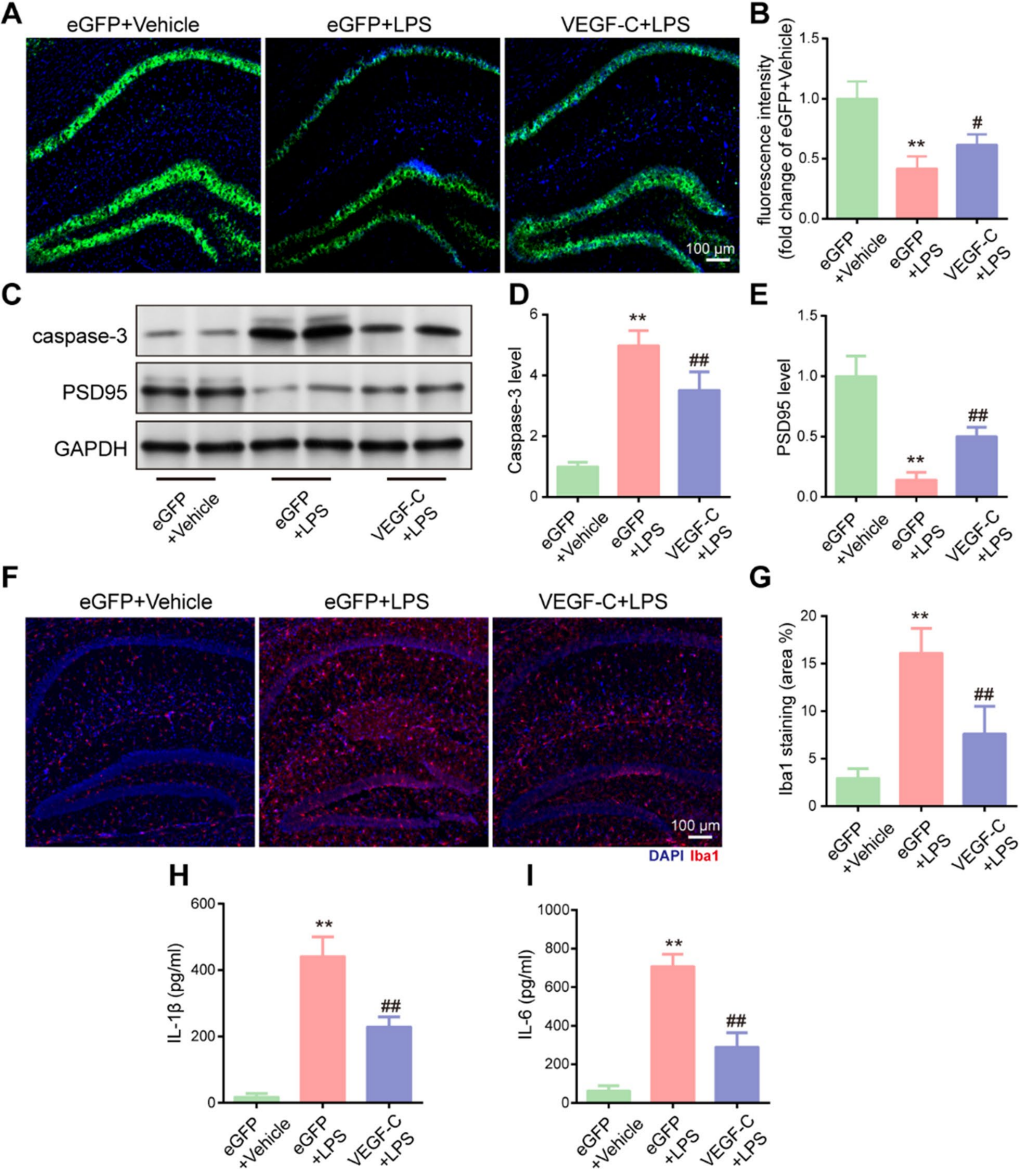
Improvement in meningeal lymphatics attenuates sepsis-induced neuronal damage and neuroinflammation in aged mice. **A** Immunofluorescence staining was used to detect NeuN (green) in the hippocampus. **B** Fluorescent intensity of NeuN in the hippocampus, n = 6. **C** The expression of caspase-3 and PSD-95 in the mouse hippocampus was detected by western blotting using specific antibodies. **D, E** Expression of caspase-3 and PSD-95 was quantified and normalized to GAPDH levels. Each value was then expressed relative to the control, which was set to 1; n = 4. **F** Immunofluorescence staining was used to detect Iba1 (red) in the hippocampus. **G** Quantitative results of the percentage of Iba1-positive area in the total area of the image, n = 6. **H**, **I** Levels of Il-1β and IL-6 in the hippocampus, n = 6. **P < 0.01, eGFP + LPS vs. eGFP + vehicle. ^#^P < 0.05, ^##^P < 0.01, VEGF-C + LPS vs. eGFP + LPS. All data are expressed as the mean ± SD

Notably, the expression of NeuN was reduced in the sepsis group; however, the levels of hippocampal neurons were obviously increased in the sepsis mice treated with VEGF-C (Fig. 5A, B). We also detected the protein levels of caspase-3 and PSD-95, a postsynaptic density protein that is closely related to neuronal function, learning, and memory, in the hippocampus of aged sepsis mice by western blotting. We found that pretreatment with VEGF-C attenuated the protein level of caspase-3 and increased the protein level of PSD-95 (Fig. 5C–E), suggesting that VEGF-C is capable protecting against sepsis-induced neuronal injury. Moreover, LPS injection induced microglial activation, which was partially reversed by pretreatment with VEGF-C in the hippocampus (Fig. 5F). As shown in the figure, VEGF-C decreased the percentage area of Iba1 coverage (Fig. 5G). The expression levels of IL-1β and IL-6 were significantly decreased in the VEGF-C group compared to those in the group treated with control viral vector (Fig. 5H, I). Collectively, these results appear to demonstrate that VEGF-C overexpression in the meninges relieves neuronal damage and neuroinflammation by facilitating meningeal lymphatic drainage and lymphangiogenesis.

### Improvement of meningeal lymphatics reduces sepsis-induced expression of disease-associated genes in aged mice

**Fig. 6:**
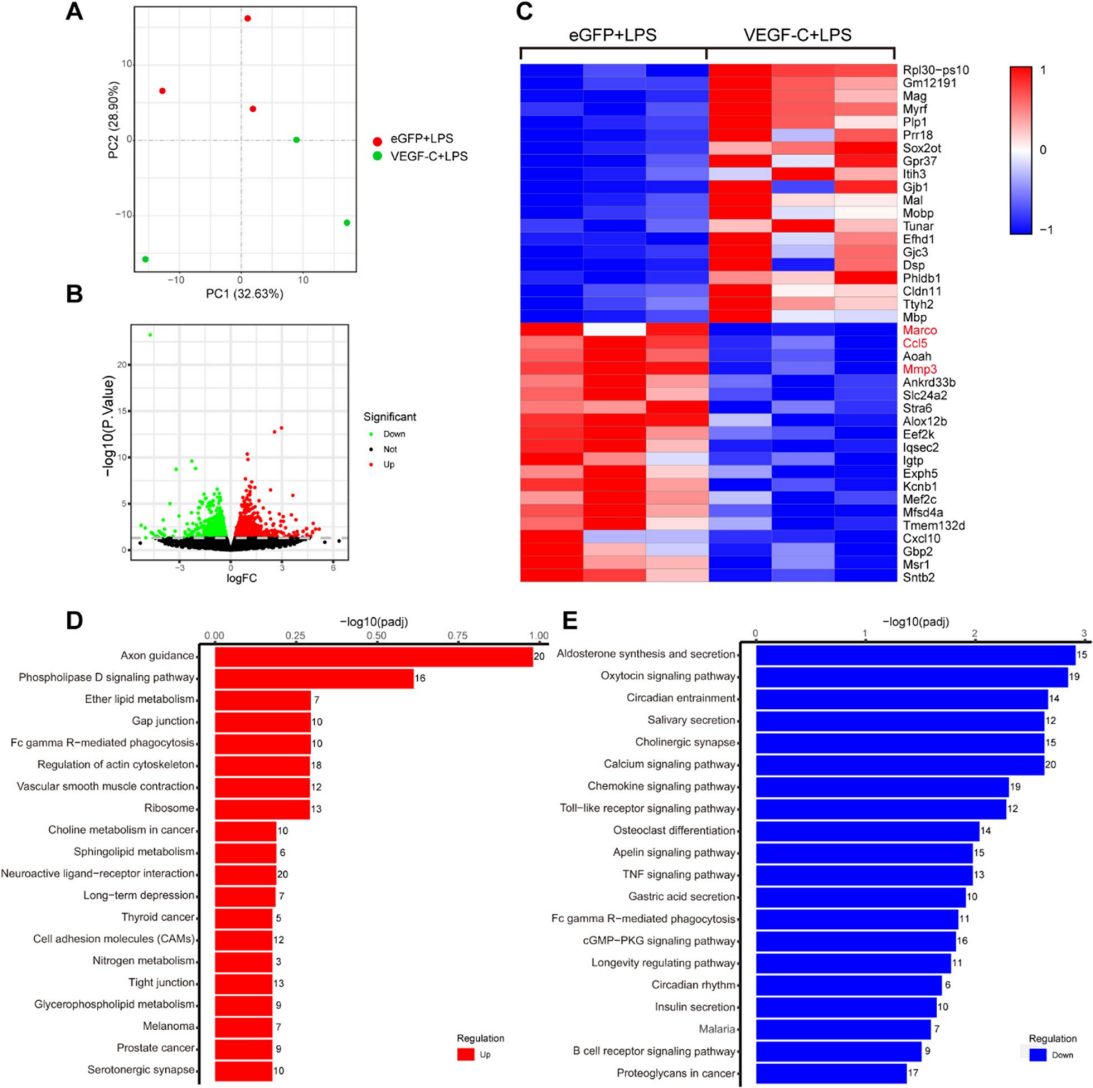
Improvement in meningeal lymphatics reduces sepsis-induced expression of disease-associated genes in aged mice. **A** Principal component (PC) analysis showing clustering of samples. **B** Volcano plots illustrate the number of significantly differentially expressed genes. **C** Heatmap representation of the top 20 most significantly upregulated and downregulated (FDR < 0.1) genes in the eGFP + LPS group vs. VEGF-C + LPS group. **D** KEGG pathway analysis of upregulated genes in the VEGF-C + LPS group compared to the eGFP + LPS group. **E** KEGG pathway analysis of downregulated genes in the VEGF-C + LPS group compared to the eGFP + LPS group

To further understand how improvement of meningeal lymphatics attenuates sepsis-induced neuronal damage and neuroinflammation, we performed transcriptome analysis of the eGFP + LPS and VEGF-C + LPS groups. The group of mice that were injected with VEGF-C + LPS clustered close together, whereas the eGFP + LPS group clustered separately (Fig. 6A). A total of 1646 differentially expressed genes (DEGs) were identified by RNA-seq analysis between the eGFP + LPS and VEGF-C + LPS groups. Furthermore, the group of aged mice treated with VEGF-C and LPS showed 898 upregulated genes and 748 downregulated genes compared to the group of aged mice treated with control eGFP and LPS (Fig. 6B). The RNA levels of the top 40 DEGs in comparisons of the eGFP + LPS and VEGF-C + LPS groups are clearly shown in the heatmap (Fig. 6C). Rlp30-ps10 was the most significantly upregulated gene. RPL30 is a ribosomal protein that has been shown to have antimicrobial function and that promotes activation of the PKC1 pathway [24]. Marco, a macrophage receptor with collagenous structure, was the most significantly downregulated gene by VEGF-C treatment. Anti-Marco treatment has been shown to increase NK cell activation and cell-mediated killing [25]. CCL5 and MMP3 were also found to be downregulated by VEGF-C treatment. CCL5 and its receptor CCR5 have been proven to impair memory linking in aged mice [26]. MMP3 is closely related to the microglia/macrophage activation state and loss of a tight junction protein (ZO-1) in the brain [27]. Gene set enrichment analysis showed that gene sets involved in the regulation of the axon guidance pathway, phospholipase D signaling pathway, ether lipid metabolism, and gap junction pathway were enriched (Fig. 6D), indicating that the function of the brain was improved by VEGF-C overexpression. Additionally, gene sets engaged in aldosterone synthesis and secretion, chemokine signaling pathway, TNF signaling pathway, and B-cell receptor signaling pathway were enriched (Fig. 6E), which indicated that VEGF-C overexpression attenuated inflammation. These findings demonstrate that SEA-induced neurological functional impairment and neuroinflammation can be attenuated by VEGF-C treatment.

### Pre-existing impairment of meningeal lymphatic drainage worsens sepsis-induced cognitive dysfunction in aged mice

As shown in Figs. 1 and 2, sepsis can severely disrupt meningeal lymphatic function. We next explored whether prior defects of meningeal lymphatic drainage in aged mice contribute to exacerbated impairment of cognitive function after sepsis and whether this may help to explain the increased severity of SAE in the elderly.

**Fig. 7:**
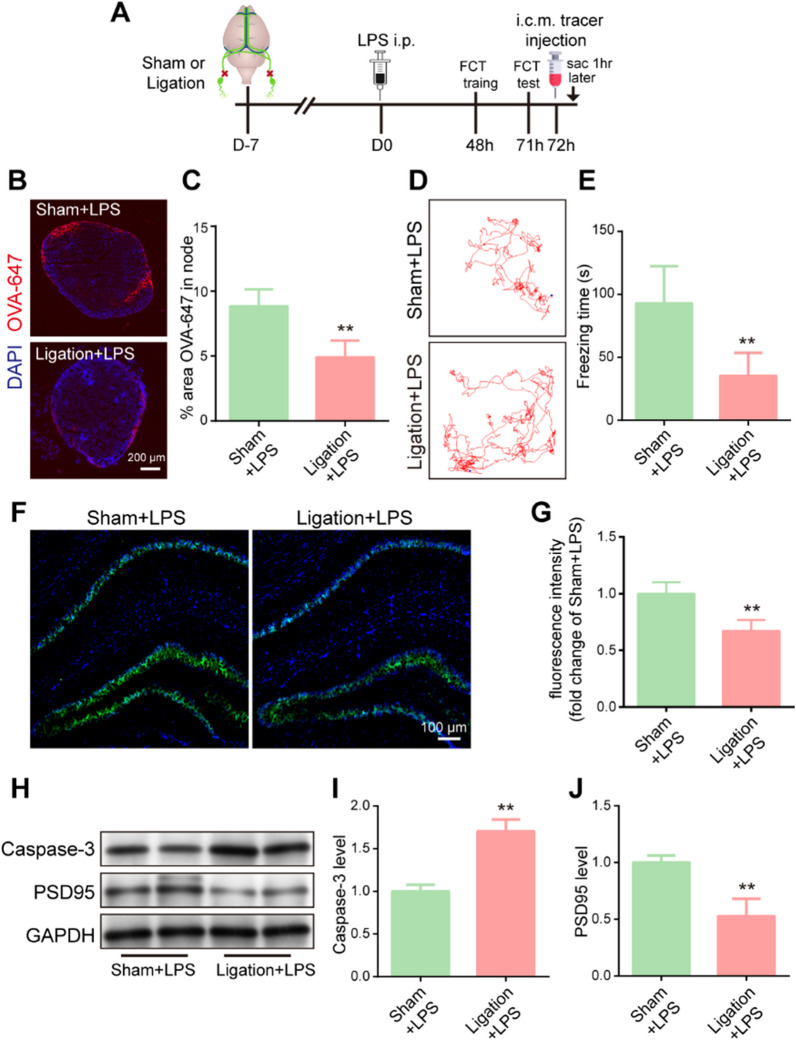
Pre-existing impairment of meningeal lymphatic drainage worsens sepsis-induced cognitive dysfunction in aged mice. **A** The experimental timeline of surgery, behavioral tests, tracer injection into the cisterna magna, and tissue collection (sac). **B** Representative immunofluorescence images of OVA-647 accumulation in dCLNs. **C** Quantification of OVA-647 accumulation in dCLNs, n = 6. **D** Representative trajectory of each group in the FCT. **E** Quantification of freezing time in the FCT, n = 8. **F** Immunofluorescence staining was used to detect NeuN (green) in the hippocampus. **G** Fluorescence intensity of NeuN in the hippocampus, n = 6. **H** The expression of caspase-3 and PSD-95 in the hippocampus was detected by western blotting using specific antibodies. **I, J** Expression of caspase-3 and PSD-95 was quantified and normalized to GAPDH levels. The ligation + LPS value was then expressed relative to the sham + LPS value, which was set to 1, n = 4**.** **P < 0.01, LPS + ligation vs. LPS + sham. All data are expressed as the mean ± SD

Therefore, to officially investigate how pre-existing impairment of the meningeal lymphatic system affects brain function after sepsis, we used a surgical method to ligate bilateral dCLNs before LPS-induced sepsis. After completing this procedure, the mice were rested for 7 days before receiving LPS treatment (Fig. 7A). After LPS injection, dCLN ligation resulted in a significant decrease in the area of the node covered by OVA-647 tracer in comparison to the sham group (Fig. 7B, C). Additionally, we showed that ligation of the dCLNs significantly damaged the cognitive function of sepsis mice (Fig. 7D, E). We then investigated NeuN expression to further examine neuronal injury in the CA1 region of the hippocampus. Unsurprisingly, ligation of the dCLNs led to a decline in NeuN expression compared to that in the sham group (Fig. 7F, G). Finally, we detected the protein levels of caspase-3 and PSD95 in the hippocampus (Fig. 7H). The level of protein caspase-3 was increased in the ligated dCLNs group (Fig. 7H, I). The ligation of dCLNs resulted in a decreased level of PSD-95 protein, indicating that dCLNs ligation may damage the synaptic function (Fig. 7H, J). In short, these data suggest that pre-existing impairment of meningeal lymphatic drainage worsens LPS-induced cognitive dysfunction and neuronal damage in aged mice.

### Pre-existing impairment of meningeal lymphatic drainage exacerbates sepsis-induced neuroinflammation in aged mice

**Fig. 8:**
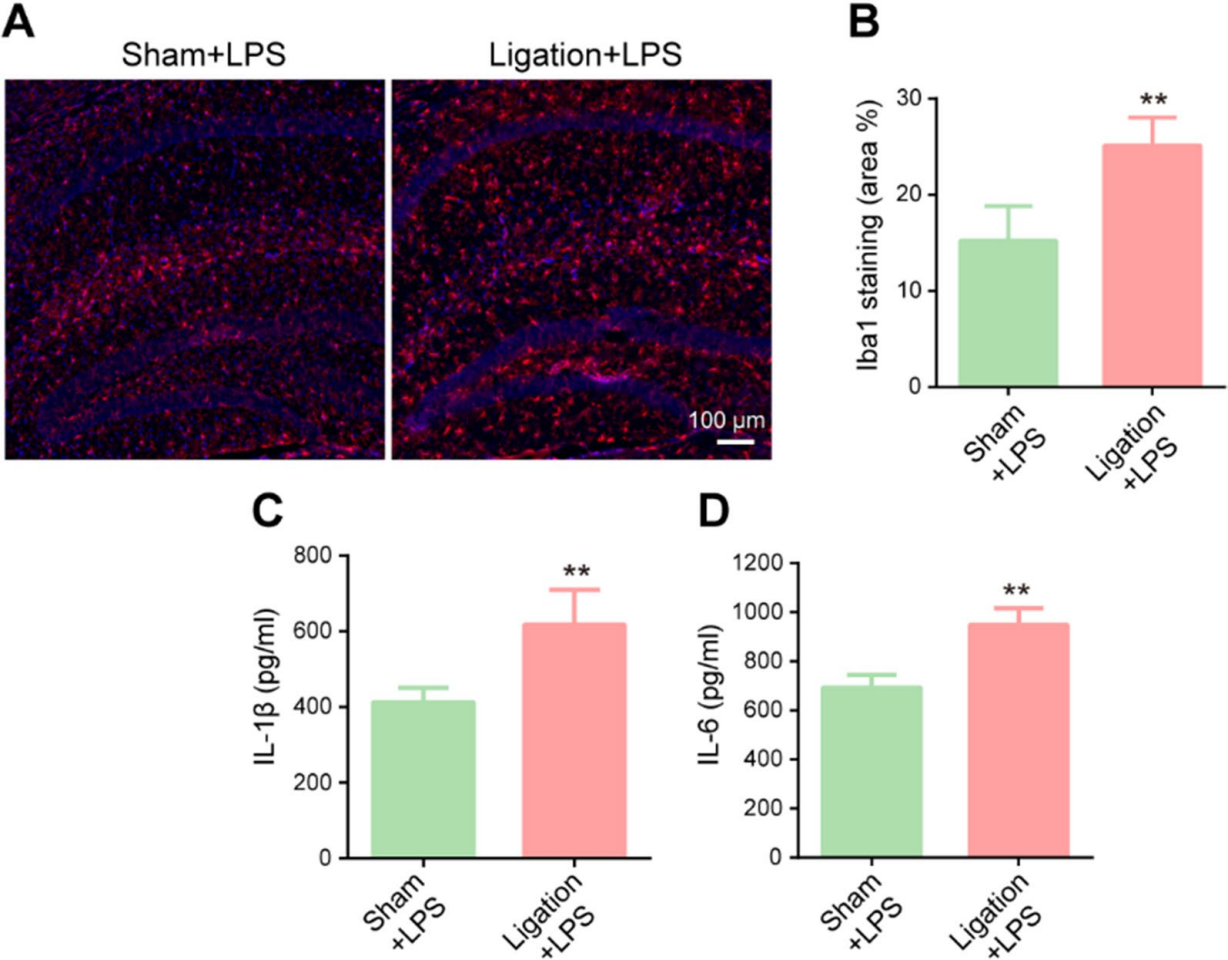
Pre-existing impairment of meningeal lymphatic drainage exacerbates sepsis-induced neuroinflammation in aged mice. **A** Immunofluorescent staining was used to detect Iba1 (red) in the hippocampus. **B** Quantitative results of the percentage of Iba1-positive areas in the total area of the image. n = 6.** C**, **D** Levels of Il-1β and IL-6 in the hippocampus, n = 6. **P < 0.01. All data are expressed as the mean ± SD

The findings from Fig. 7 motivated us to explore whether sepsis mice with pre-existing meningeal lymphatic dysfunction show more serious neuroinflammation. To verify the severity of the inflammation, we measured the levels of Iba1 and inflammatory factors. We found that ligated dCLNs resulted in a higher percentage area of Iba1 in the hippocampus (Fig. 8A, B). Furthermore, the ligated + LPS group had higher levels of IL-1β and IL-6 than the LPS group (Fig. 8C, D). Taken together, these data suggest that neuroinflammation may be exacerbated if SAE occurs in a setting of pre-existing meningeal lymphatic dysfunction.

## Discussion

The relationship between SAE and meningeal lymphatic vessels is poorly understood. Our study has shown the importance of the meningeal lymphatic system for SAE therapy. We found that meningeal lymphatic function is promptly damaged following sepsis and that it may take a while to completely return to normal levels. Furthermore, we demonstrated that the delivery of AAV1–VEGF-C through the magna cisterna improves the drainage of meningeal lymphatics in mice with sepsis. AAV1–VEGF-C treatment can also attenuate microglial activation and neuroinflammation and alleviate sepsis-induced cognitive impairment. We also showed that pre-existing meningeal lymphatic dysfunction by ligating dCLNs aggravated sepsis-induced neuroinflammation and cognitive deficits. Collectively, these findings reveal a functional role of meningeal lymphatics in mediating neuroinflammation in sepsis and provide a new modality of SAE therapy.

SAE causes long-term cognitive impairment and deteriorates the neuroinflammatory process with immune dysfunction, particularly in the aging community [28]. The pathological features of SAE are complex processes of dysregulated immune response and systemic inflammation [29, 30]. Various factors are reportedly involved in the nosogenesis of SAE, including inflammatory cytokines, BBB breakdown, ischemic processes, deterioration of brain homeostasis, and decline in metabolism [31], although the precise mechanism is still unknown. The existing mechanisms of SAE largely focus on activating the brain’s immune system. However, how inflammation develops and worsens, leading to the deterioration of homeostasis in the brain and causing sepsis in elderly patients, remains to be studied.

Since the discovery of meningeal lymphatics in 2015, an increasing body of evidence has demonstrated that the meningeal lymphatic system plays a key role in regulating immune responses and inflammation in the central nervous system [11, 13, 14, 32–34]. In these studies, increased drainage of the meningeal lymphatic system resulted in a better response to systemic immunotherapy against brain tumors and neuroinflammation after traumatic brain injury [32, 35], as well as ameliorated cognition and memory performance in age-associated neurodegenerative diseases such as Alzheimer’s disease [36, 37]. It is unknown whether meningeal lymphatic drainage is engaged in facilitating or alleviating neuroinflammation and whether it is directed against specific disease conditions. For instance, several studies have found that lymphatic drainage plays a role in stimulating autoimmunity, as it promotes the drainage of brain antigens into dCLNs [38, 39]. In experimental autoimmune encephalomyelitis (EAE), ablation of meningeal lymphatic drainage ameliorates EAE (spontaneous) pathology by limiting the drainage of MOG into dCLNs, thereby preventing the activation of MOG-specific T cells [33]. In other circumstances, the meningeal lymphatic system serves as a drain for macromolecules, protein aggregates, and even triacylglycerols of the CNS and works as a “cleaner” for the waste of the CNS [40]. In a mouse model of Alzheimer’s disease [11], ablation of lymphatic drainage using visudyne photoablation or lymphatic ligation led to deposition of amyloid-β in the meninges and hippocampus. Furthermore, recent studies have indicated that VEGF-C-driven meningeal lymphatic drainage is essential for modulating depression-like behavior and generating an efficient immune response against brain tumors [21, 35, 40].

However, the role of the meningeal lymphatic system in SAE is not fully understood, especially in the aged. Here, we confirmed that sepsis can rapidly impair the drainage of meningeal lymphatic vasculature and found that the damage to lymphatic drainage is more severe in aged mice, which may explain the more severe effects of SAE on brain function and neuroinflammation in elderly people. Our data also suggest that sepsis reduces the coverage of the meningeal lymphatic vasculature, which may alter CSF drainage, resulting in a reduction in CSF reaching the hot spots of the meningeal lymphatics. Such alterations in CSF drainage may be attributed to the morphological impairment of meningeal lymphatics. We also found that overexpression of VEGF-C increased meningeal lymphangiogenesis and enhanced lymphatic drainage, leading to the improvement of neuroinflammation and cognitive impairment in SAE. In a mouse model of hepatic encephalopathy (HE), VEGF-C overexpression was found to attenuate brain inflammation and restore motor function in bile duct ligation rats [41]. These results indicate that meningeal lymphangiogenesis induced by VEGF-C ameliorates neuroinflammation and gliosis by facilitating lymphatic drainage and improving neurological diseases.

Aging is a major risk factor for many neurodegenerative diseases, including Alzheimer’s disease [42]. Indeed, Da Mesquita et al. demonstrated that lymphatic drainage of aged mice was impaired, which was accompanied by a decrease in the diameter and coverage of meningeal lymphatic vessels, as well as a decrease in CSF macromolecules into dCLNs [11]. Several studies have also suggested that lymphatic drainage function is impaired in the aging human brain, as examined by magnetic resonance imaging (MRI) [15, 43, 44]. Moreover, as shown in Fig. 3, sepsis can cause significant impairment of meningeal lymphatic drainage in aged mice compared with young mice. These findings led us to hypothesize that the deterioration of meningeal lymphatic vessels may be the main reason for the vulnerability of the elderly to SAE after sepsis. Therefore, we ligated the dCLNs before LPS injection to block lymphatic drainage. Previous lymphatic drainage defects worsen sepsis-induced neuroinflammation and cognitive dysfunction, further explaining why the elderly are more likely to exhibit activation of microglia and grievous neuroinflammation than young people when faced with sepsis.

Neuroinflammation plays a crucial role in the pathogenesis of SAE and often persists for months or even years in the elderly [2, 45]. Based on previous research, impaired drainage of DAMPs (e.g., amyloid β, cellular debris, necrotic cells, and other macromolecules) from the brain may trigger long-term immune activation in the injured brain [46]. It has been proven that timely and effective intervention against neuroinflammation has positive effects on cognitive impairment caused by SAE [47]. However, the mechanism of uncontrolled inflammatory responses during SAE, which leads to cognitive dysfunction, remains difficult to determine. Activated microglia can release many cytokines, including IL-1β, which is negatively correlated with the severity of memory dysfunction [48]. Our results revealed that enhanced lymphatic drainage suppressed the activation of microglia and the expression of inflammatory factors and increased the level of PSD95 in SAE, suggesting that VEGF-C treatment is of great significance for hippocampal neuron activity, synaptic plasticity, and early prevention of SAE.

We explored the potential mechanism by which enhanced meningeal lymphatic drainage alleviates neuroinflammation in SAE by RNA-seq analysis. We identified genes that were significantly changed in SAE but reversed by VEGF-C overexpression. Among them, MYRF is a transcriptional regulator, the upregulation of which is necessary for oligodendrocyte differentiation and myelin maintenance [49]. In the latter study, the phosphorylation of MYRF mediated demyelination and induced chronic inflammation in multiple sclerosis [50]. Under these conditions, the knockdown of MYRF has been shown to impair some behaviors, including learning complex wheel running [51] and spatial memory [52]. In our RNA-seq analysis of the hippocampus, we observed an obvious decline in MYRF expression in eGFP-treated mice. However, this decline can be restored by VEGF-C overexpression, which indicates that enhanced lymphatic drainage induced by VEGF-C promotes oligodendrocyte differentiation and myelin maintenance, resulting in improvements in spatial memory and chronic inflammation. We also observed that VEGF-C treatment suppressed oxytocin signaling, chemokine signaling, Toll-like receptor signaling, TNF signaling, and the B-cell receptor signaling pathway, which indicates that neuroinflammation is inhibited by VEGF-C pretreatment. Therefore, promoting functional recovery of the meningeal lymphatic system may represent a novel therapeutic target for SAE. However, the mechanism underlying meningeal lymphatic disruption caused by sepsis is unclear. Da Mesquita et al. showed that a reduction in CCR7 expression by meningeal T cells in old mice is linked to worsened glymphatic function and neuroinflammation [53]. Further studies are needed to explore whether meningeal immune cells are involved in sepsis-induced meningeal lymphatic dysfunction.

Several studies have reported the drainage of CSF to extracranial lymph nodes via the perineural and perivascular subarachnoid spaces of cranial nerves and cranial arteries [54, 55]. Considering that, visudyne, a photoconvertible drug, or AAV virus, could be employed in subsequent studies to selectively ablate meningeal lymphatic vasculature and elucidate its role in SAE.

## Conclusions

In conclusion, the results described here reveal that the meningeal lymphatic system is damaged in sepsis, and pre-existing defects in this drainage system intensify the neuroinflammation and cognitive dysfunction induced by SAE. Meningeal lymphatic drainage may become incapable of clearing increased DAMPs in the brain in SAE. More importantly, we provide evidence that increased meningeal lymphatic system activity induced by VEGF-C overexpression effectively reduces sepsis-induced neuroinflammation and cognitive dysfunction, which provides a promising target for the prevention and treatment of SAE in elderly patients.

### Supplementary Information

**Additional file 1: Table.** The number of animals used for multiple study parameters. **Figure S1**. Open field was performed to analyze the locomotor activity at day 3 after LPS injection. **Figure S2**. Intracisternal injection of the AAV1-eGFP virus into the cisterna magna did not yield any observable effect on liver lymphatic lymphangiogenesis. **Figure S3**. Schematic diagram of lymph fluid from the head entering the blood circulation.

## Data Availability

The RNA sequencing data generated for this study can be found in the GEO repository under accession number GSE247222. The datasets generated during this study are available from the corresponding author upon reasonable request.

## Abbreviations

AAV1: Adeno-associated virus 1
BBB: Blood–brain barrier
CCL5: C–C motif chemokine 5
CNS: Central nervous system
dCLNs: Deep cervical lymph nodes
ELISA: Enzyme-linked immunosorbent assay
FCT: Fear conditioning test
FACS: Fluorescence-activated cell sorting
IL-1β: Interleukin-1β
IL-6: Interleukin-6
Iba1: Ionized calcium-binding adapter molecule 1
LPS: Lipopolysaccharide
Lyve-1: Lymphatic vessel endothelial receptor-1
MMP3: Matrix metalloproteinase 3
PSD-95: Postsynaptic density protein-95
qRT-PCR: Quantitative real-time PCR
SAE: Sepsis-associated encephalopathy
VEGF-C: Vascular endothelial growth factor C
