# Supplementary material for: Enhanced meningeal lymphatic drainage ameliorates lipopolysaccharide-induced brain injury in aged mice
Source: J Neuroinflammation. 2024 Jan 30;21:36. doi: 10.1186/s12974-024-03028-4 (PMC10826026; doi:10.1186/s12974-024-03028-4)
Supplement: Supplementary file 1 — Additional file 1: Table. The number of animals used for multiple study parameters. Figure S1. Open field was performed to analyze the locomotor activity at day 3 after LPS injection. Figure S2. Intracisternal injection of the AAV1-eGFP virus into the cisterna magna did not yield any observable effect on liver lymphatic lymphangiogenesis. Figure S3. Schematic diagram of lymph fluid from the head entering the blood circulation. [file 12974_2024_3028_MOESM1_ESM.docx]

**Table. The number of animals used for multiple study parameters**

Part 1. Sepsis impairs meningeal lymphatic drainage function in aged mice

| Group | Immunofluorescence staining | Western blot | Sorting of meningeal LECs |
| --- | --- | --- | --- |
| Vehicle | 6 | 4 | 12 |
| LPS 1d | 6 | 4 | 12 |
| LPS 3d | 6 | 4 | 0 |
| LPS 7d | 6 | 4 | 0 |

Part 2. Meningeal lymphatic function of aged mice is more vulnerable to sepsis

| Group | Immunofluorescence staining |
| --- | --- |
| Aged + Vehicle | 5 |
| Aged + LPS | 5 |
| Young + LPS | 5 |

Part 3.窗体顶端

Identification of the infection effects of AAV1-eGFP on the meninges

| Group | Immunofluorescence staining |
| --- | --- |
| Vehicle | 3 |
| AAV1-eGFP | 3 |

Part 4. Improvement of meningeal lymphatics alleviates sepsis-induced cognitive dysfunction and neuroinflammation in aged mice

| Group | Immunofluorescence staining | Behavioral testing | Western blot | ELISA | Transcriptome analysis |
| --- | --- | --- | --- | --- | --- |
| eGFP +LPS | 6 | 8 | 4 | 6 | 3 |
| VEGF-C + LPS | 6 | 8 | 4 | 6 | 3 |

Part 5. Pre-existing impairment of meningeal lymphatic drainage worsen sepsis-induced cognitive dysfunction and neuroinflammation in aged mice

| Group | Immunofluorescence staining | Behavioral testing | Western blot | ELISA |
| --- | --- | --- | --- | --- |
| Sham + LPS | 6 | 8 | 4 | 6 |
| Ligation + LPS | 6 | 8 | 4 | 6 |


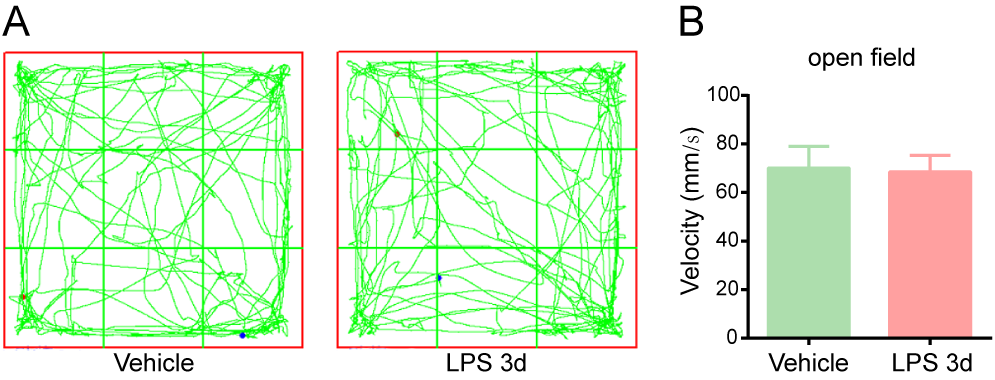


**Figure S1. Open field was performed to analyze the locomotor activity at day 3 after LPS injection.** **A**. Representative trajectory of each group in the open field test. **B**. Quantification of velocity in the open field test, n = 8, mean ± SD.


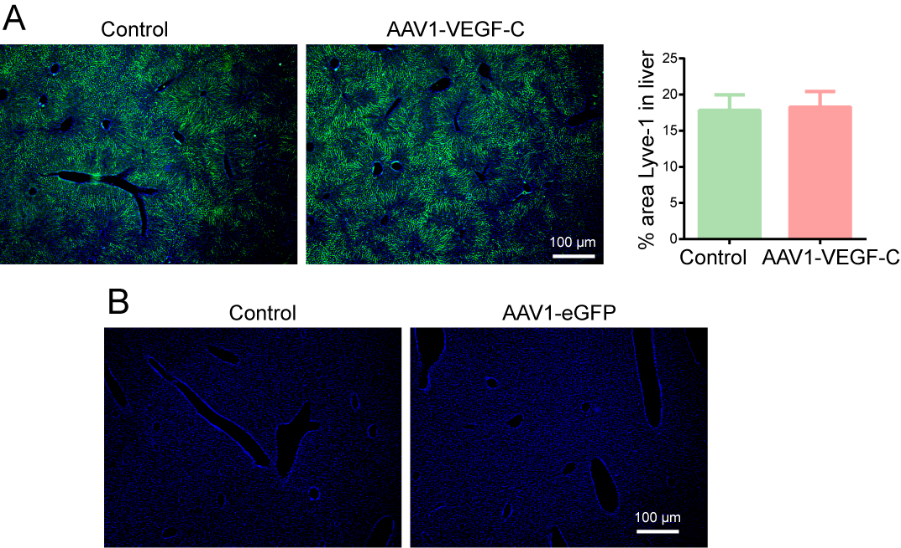


**Figure S2. Intracisternal injection of the AAV1 virus into the cisterna magna did not yield any observable effect on liver lymphatic lymphangiogenesis. A.** Representative immunofluorescence images of liver stained with Lyve-1 and Graph depicting the percentage area of Lyve-1 coverage in the liver. **B.** Expression of eGFP in the liver.


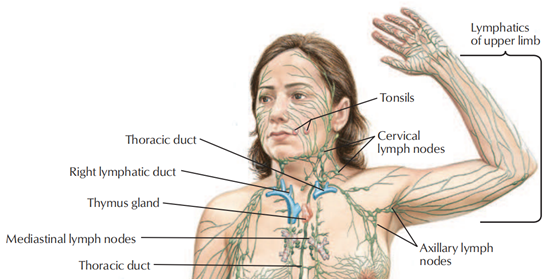


**Figure S3. Schematic diagram of lymph fluid from the head entering the blood circulation.** Lymph fluid originates from the head, particularly the meninges. The meningeal lymphatic vessels transport the lymph fluid to the cervical lymph nodes. From the cervical lymph nodes, the lymph fluid converges toward the venous angle. The lymph fluid is pumped by the heart and circulates through the arterial system, reaching various parts of the body. Netter's atlas of human anatomy, 2017.7e.
